# PredictProtein—an open resource for online prediction of protein structural and functional features

**DOI:** 10.1093/nar/gku366

**Published:** 2014-05-05

**Authors:** Guy Yachdav, Edda Kloppmann, Laszlo Kajan, Maximilian Hecht, Tatyana Goldberg, Tobias Hamp, Peter Hönigschmid, Andrea Schafferhans, Manfred Roos, Michael Bernhofer, Lothar Richter, Haim Ashkenazy, Marco Punta, Avner Schlessinger, Yana Bromberg, Reinhard Schneider, Gerrit Vriend, Chris Sander, Nir Ben-Tal, Burkhard Rost

**Affiliations:** 1 Department of Informatics, Bioinformatics & Computational Biology i12, TUM (Technische Universität München), Garching/Munich 85748, Germany; 2 Biosof LLC, New York, NY 10001, USA; 3 TUM Graduate School, Center of Doctoral Studies in Informatics and its Applications (CeDoSIA), TUM (Technische Universität München), Garching/Munich 85748, Germany; 4 New York Consortium on Membrane Protein Structure (NYCOMPS), Columbia University, New York, NY 10032, USA; 5 Department of Genome Oriented Bioinformatics, Technische Universität München, Wissenschaftszentrum Weihenstephan, Freising 85354, Germany; 6 The Department of Cell Research and Immunology, George S. Wise Faculty of Life Sciences, Tel Aviv University, 69978 Tel Aviv, Israel; 7 Wellcome Trust Sanger Institute, Hinxton, Cambridgeshire, CB10 1SA, UK; 8 European Molecular Biology Laboratory, European Bioinformatics Institute, Hinxton, Cambridgeshire, CB10 1SD, UK; 9 Department of Pharmacology and Systems Therapeutics, Icahn School of Medicine at Mount Sinai, One Gustave L. Levy Place, New York, NY 10029, USA; 10 Department of Biochemistry and Microbiology, Rutgers University, New Brunswick, NJ 08901, USA; 11 Luxembourg University & Luxembourg Centre for Systems Biomedicine, 4362 Belval, Luxembourg; 12 CMBI, NCMLS, Radboudumc Nijmegen Medical Centre, 6525 GA Nijmegen, The Netherlands; 13 Computational Biology Program, Memorial Sloan Kettering Cancer Center, New York, 10065 NY, USA; 14 The Department of Biochemistry and Molecular Biology, George S. Wise Faculty of Life Sciences, Tel Aviv University, 69978 Tel Aviv, Israel; 15 Department of Biochemistry and Molecular Biophysics & New York Consortium on Membrane Protein Structure (NYCOMPS), Columbia University, New York, NY 10032, USA; 16 Institute for Advanced Study (TUM-IAS), Garching/Munich 85748, Germany; 17 Institute for Food and Plant Sciences WZW-Weihenstephan, Alte Akademie 8, Freising 85350, Germany

## Abstract

PredictProtein is a meta-service for sequence analysis that has been predicting structural and functional features of proteins since 1992. Queried with a protein sequence it returns: multiple sequence alignments, predicted aspects of structure (secondary structure, solvent accessibility, transmembrane helices (TMSEG) and strands, coiled-coil regions, disulfide bonds and disordered regions) and function. The service incorporates analysis methods for the identification of functional regions (ConSurf), homology-based inference of Gene Ontology terms (metastudent), comprehensive subcellular localization prediction (LocTree3), protein–protein binding sites (ISIS2), protein–polynucleotide binding sites (SomeNA) and predictions of the effect of point mutations (non-synonymous SNPs) on protein function (SNAP2). Our goal has always been to develop a system optimized to meet the demands of experimentalists not highly experienced in bioinformatics. To this end, the PredictProtein results are presented as both text and a series of intuitive, interactive and visually appealing figures. The web server and sources are available at http://ppopen.rostlab.org.

## INTRODUCTION

Molecular biology is moving into the high-throughput mode as the number of experiments needed to support a single hypothesis is rapidly growing. The line between experimental result and computational analysis is blurring; this also shifts what constitutes a reliable annotation. On top, the vast amount of life science data outpaces computer power. For example, less than 1% of the over 51 million sequences in UniProt (February 2014) (1) have some expert annotations in Swiss-Prot. This protein annotation gap widens every day (2). PredictProtein is one of the resources applicable to all proteins that contribute to closing this gap. 

The PredictProtein (PP) server is an automatic service that searches up-to-date public sequence databases, creates alignments, and predicts aspects of protein structure and function. In 1992, PredictProtein went online as one of the first Internet servers in molecular biology at the EMBL (Heidelberg, Germany). From 1999 to 2009, the server operated from Columbia University (New York, NY) and in 2009 it moved to the TUM (Munich, Germany). PredictProtein was one of the first services realizing state-of-the-art protein sequence analysis, and the prediction of structural and functional features in a single server. While many outstanding services (3) have expanded on some of those aspects, PredictProtein has remained one of the most comprehensive resources. The thousands of citations to PredictProtein and to our methods demonstrate the server's applicability and acceptance. Since 2009, for example, its website was visited more than one million times by about 80 000 unique visitors per year from 139 countries. Furthermore, over 500 000 sequences were submitted and processed by the service. About half of all submitted sequences were not in UniProt (1) at the time of submission. This suggests that the server's primary utility is in providing annotations for uncharacterized proteins. The following two central principles have guided the evolution of PredictProtein.

1. *Sustained quality with performance estimates.* The performance of many tools is not sufficiently assessed and/or their performance does not sustain over time. Two decades of Critical Assessment of protein Structure Prediction (CASP)-like experiments (4,5) have demonstrated this repeatedly. PredictProtein went online with a method for the prediction of protein secondary structure (PHD (6)) and 22 years later the performance estimates for that method continue to be valid: a unique achievement.
2. *Ease of use.* From the beginning we have aspired to make the use of our tools intuitive for all users. Unfortunately, the growth in size and scope continues to challenge the realization of this guiding principle. In 1992, the service provided alignments and secondary structure prediction; in 2014, it includes over 30 complex tools. Creating a unified, natural interface for these tools is challenging. Furthermore, we need to invest more resources to sustain the increasing usage as the data flood surges on. For example, most of our CPU goes into running PSI-BLAST (7). Since 2009, databases grew 10-fold whereas the CPU speed has only tripled, i.e. we need at least three times the number of CPUs we currently have to achieve the same ease in handling each job.

## METHODS

### PredictProtein incorporates over 30 tools

Supplementary Table S1, Supporting Online Material provides a comprehensive list of all components. *Database searches:* sequences similar to the query are identified by standard, pairwise BLAST (8) and iterated PSI-BLAST (7) searches (9,10) against a non-redundant combination of PDB (11), Swiss-Prot (12) and TrEMBL (1). In addition, functional motifs are taken from PROSITE (13) and domains from Pfam (14). *Prediction of structural features:* predicted aspects of structure include PROFphd secondary structure and solvent accessibility (15,16), PROFtmb transmembrane strands (17), TMSEG transmembrane helices, COILS coiled-coil regions (18), DISULFIND disulfide bonds (19) and SEG low-complexity regions (20). Disordered regions are predicted by a set of tools: UCON (21), NORSnet (22), PROFbval (23,24) and Meta-Disorder (25). *Prediction of functional features:* predicted aspects include ConSurf annotations and visualizations of functionally important sites (26,27), protein mutability landscape analysis showing the effect of point mutations on protein function predicted by SNAP2 (28), Gene Ontology (GO) terms from metastudent (29), LocTree3 predictions of subcellular localization (30), protein–protein interaction sites (ISIS2) and protein–DNA, protein–RNA binding sites (SomeNA). Almost all prediction methods use evolutionary information obtained from PSI-BLAST searches; the more related protein sequences are found and the more divergent those are, the higher the gain in performance (10,15). However, none of the methods (with the exception of metastudent, see below) relies solely on profiles and the prediction without a profile is significantly better than random. For most prediction methods (e.g. LocTree3 and SNAP2) the prediction quality is estimated by a reliability score. In the following, we introduce some of the recent and upcoming additions since 2004 (31) in more detail.

### New: TMSEG transmembrane helix predictions

TMSEG (Bernhofer, M. *et al.*, in preparation) predicts alpha-helical transmembrane proteins, the position of transmembrane helices, and membrane topology. The method uses a novel segment-based neural network to refine the final prediction. TMSEG was developed and evaluated on 166 transmembrane proteins extracted from PDBTM (32) and OPM (33), and on 1441 proteins from the SignalP4.1 dataset (34). In our hands, TMSEG appears to complement and improve over the best existing methods (e.g. PolyPhobius (35) and Memsat3 (36)) predicting all membrane helices correctly for about 60% of all proteins. The method correctly identifies 98% of all transmembrane proteins with a false positive rate of less than 2%.

### New: SNAP2 predict effect of mutations upon function

**Figure 1.**
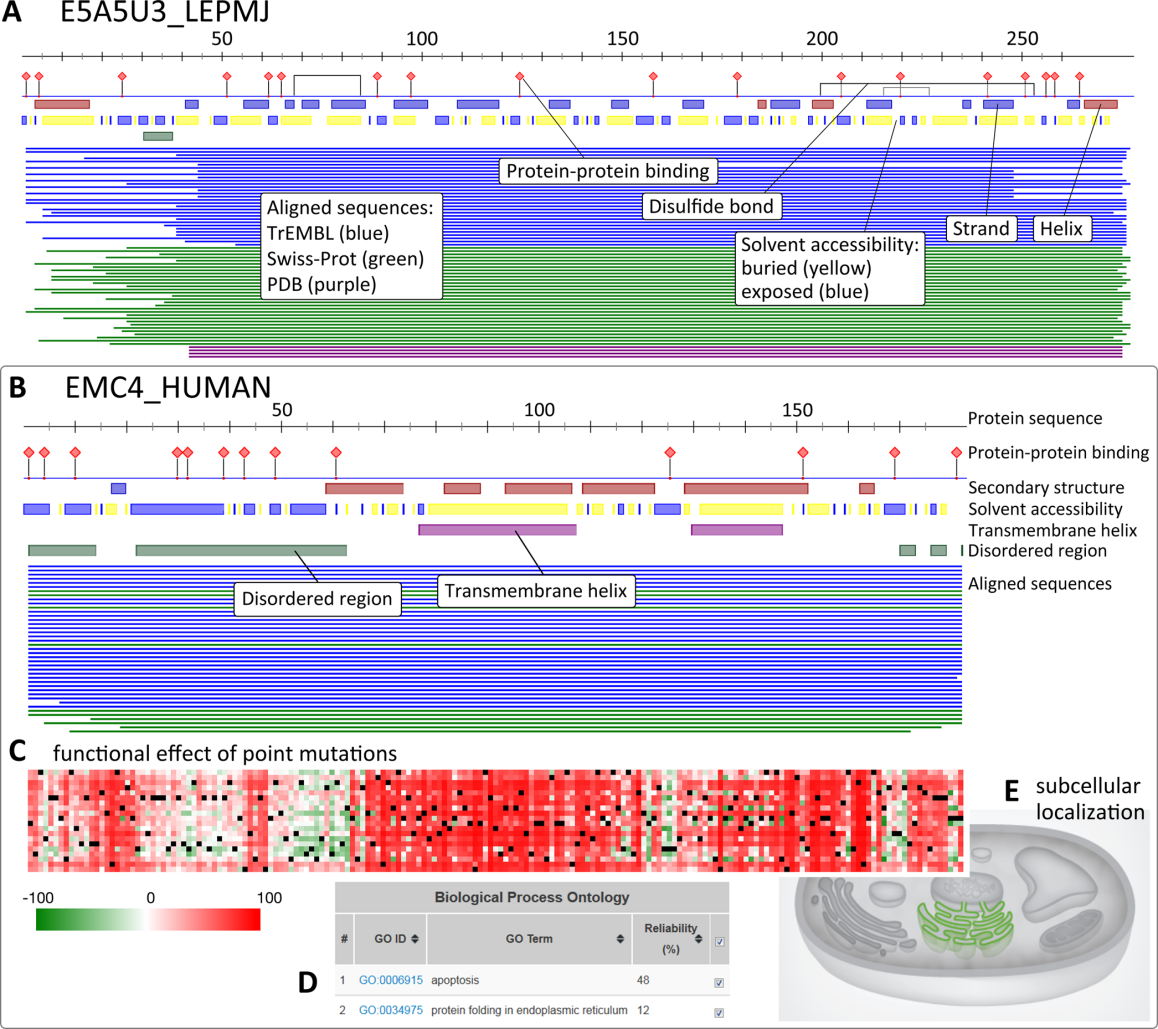
Visual results from PredictProtein (PP). The PP Dashboard Viewer shows a schematic of all position-based predictions and sequence alignments. (**A**) Putative protein (UniProt AC E5A5U3). (**B**) ER membrane protein complex subunit 4 (EMC4, UniProt AC Q5J8M3). The protein sequence is represented by a scale on top of the predicted features. Features presented include protein–protein binding sites (ISIS2), disulfide bonds (DISULFIND), structural features such as secondary structure state and solvent accessibility (PROFphd), transmembrane helices (TMSEG) and disordered regions (MD). Proteins aligned by PSI-BLAST (7) are shown as thin lines colored by database origin (PDB (11), Swiss-Prot (12) and TrEMBL (1)). Clicking on each line links to the database entry of the hit. For all elements, tooltips disclose the annotated feature, its position in the sequence and its type (prediction versus database search). (**C**) A complete analysis of the functional effect of point mutations on EMC4 shown in a heatmap (SNAP2). (**D**) Predicted GO terms (metastudent) for EMC4 in tabular format. (**E**) The predicted cellular compartment, ER membrane, for EMC4 (LocTree3) is highlighted in green in a schematic of a eukaryotic cell.

SNAP2 predicts the effect of single amino acid substitutions on protein function (37). It improves over its predecessor SNAP (38) by using additional coarse-grained features that better classify samples with unclear evidence. With a two-state accuracy of 83% and an AUC of 0.91, SNAP2 performs on par or better than other state-of-the-art methods on human variants while significantly outperforming these methods for other organisms. SNAP2 is the only available method predicting the effect of point mutations even without alignment information (if fewer than 10 related proteins are found, a specific method is applied with an expected accuracy of ∼70% instead of 83%). For each protein we also predict the entire protein mutability landscape (28,39), i.e. the functional effect of all possible point mutations. The results are displayed in a heatmap representation (40) of functional effects (Figure 1C).

### New: LocTree3 subcellular localization for all domains of life

LocTree3 predicts subcellular localization for proteins in all domains of life (30). The method predicts the localization in 18 classes (8 classes for transmembrane and 10 classes for soluble proteins) for eukaryotes, in 6 for bacteria and in 3 for archaea. LocTree3 successfully combines de novo (41) and homology-based predictions (7), reaching an 18-state prediction accuracy over 80% for eukaryotes and a 6-state accuracy over 89% for bacteria. The high level of performance and the large number of predicted classes make LocTree3 the most comprehensive and most accurate tool for subcellular localization prediction.

### New: metastudent infers GO terms by homology

The method metastudent (29) predicts GO (42) terms through homology inference. It first BLASTs queries against proteins with experimental GO annotations taken from Swiss-Prot (12), i.e. when no hit to any protein with experimentally annotated GO term is returned, no prediction is made. Then, three algorithms independently choose which GO terms to inherit. These differ in the amount and quality of alignment hits considered and how they assign a probability to each GO term. A meta-classifier combines the three through linear regression. metastudent achieves a maximum F1 score of 0.36 in the biological process ontology and of 0.48 in the molecular function ontology (29). Although this is slightly worse (within the error estimates (43)) than the best method for predicting GO terms (44), the advantage is that metastudent predictions can easily be traced back to the experimental annotations upon which they are based.

### Recent: Meta-Disorder prediction of protein disorder

Intrinsically disordered or unstructured regions in proteins do not fold into well-defined three-dimensional (3D) structures when in isolation, but may become structured upon binding to a substrate. Because of the heterogeneity of disordered regions, we have developed several methods predicting different types of disorders. UCON (21) combines protein-specific pairwise contacts predicted by PROFcon (45) with pairwise statistical potentials to predict long disordered regions that are rendered intrinsically unstructured by few internal connections. NORSnet (22) predicts disordered regions with NO Regular Secondary structure (NORS (46), i.e. long loops), separating very long disordered loops predicted by NORSp (47) from all other regions in the PDB (11). PROFbval (23,24), trained on B-values in X-ray structures, predicts flexible residues in short disordered regions. Meta-Disorder (25) is a neural-network-based meta-predictor that uses different sources of information, including the orthogonal disorder predictors mentioned above and others, e.g. IUPred (48) and DISOPRED (49). Meta-Disorder significantly outperforms its constituents (25,50). A comprehensive, independent study (50), on disordered regions from the PDB and DisProt (51), suggested Meta-Disorder to be one of the top two methods available.

### Recent: protein–protein binding sites

Residues that can bind other proteins are now predicted by ISIS2 instead of ISIS (52). ISIS splits a query sequence into windows of nine consecutive residues, encoding each window as a vector of features (e.g. PSI-BLAST amino acid conservation frequencies or predicted secondary structure). A neural network, trained on existing protein–protein binding residue annotations, determines whether a query residue can bind other proteins. ISIS2 has been trained on a large dataset of PDB-annotated binding sites (53). A faster neural network implementation (53) and new methods for predicting residue features further improve the accuracy of ISIS2.

### Recent: protein–DNA, protein–RNA binding sites

Protein–polynucleotide binding underlies important processes such as replication and transcription. SomeNA (54) predicts protein–polynucleotide binding on three levels. First, it predicts which proteins bind nucleotides. Second, it predicts the type of binding (RNA or DNA or both). Third, it predicts the protein residues that bind DNA or RNA. The first step is performed best: 77% of the proteins are correctly predicted to bind DNA and RNA. The distinction between the type of nucleotide is slightly more difficult: 74% of the proteins predicted to bind DNA and 72% of the proteins predicted to bind RNA were correct. Slightly over 53% of the residues binding DNA and/or RNA were correctly predicted. These levels of performance are at least 3-fold higher than random.

### Recent: ConSurf conservation of surfaces explains function

ConSurf (26,27) estimates the evolutionary rate in protein families. These rates are useful for protein structure and function prediction because they reflect constrains imposed on the general evolutionary drift (10,15,55). Queried with a protein sequence, ConSurf first finds related sequences in UniProt (1). Evolutionary rates of amino acids are estimated based on evolutionary relatedness between the protein and its homologues using either empirical Bayesian (56) or maximum likelihood (57) methods. The strength of these methods is that they rely on the phylogeny of the sequences and thus can accurately distinguish between conservation due to short evolutionary time and conservation resulting from importance for maintaining protein foldability and function. If a structure is available, ConSurf maps the patterns of conservation upon the 3D structure. These patterns reveal crucial details about protein function.

## WEB SERVER—UPDATES AND SOFTWARE

### Graphical front-end

The dashboard page of PredictProtein results uses the BioJS (58) FeatureViewer component to show protein features (Figure 1A and B). Along the protein sequence, features are indicated by color and single residue pins. Depending on the protein, the overview features may include predictions of secondary structure and solvent accessibility, transmembrane helices, disulfide bonds and disordered regions. Details are available by zooming-in on local regions. Other views present additional annotations and predictions, e.g. functional landscapes of the effect of point mutations (SNAP2, Figure 1C), predicted GO terms (metastudent, Figure 1D) or subcellular localization (LocTree3, Figure 1E). In the dashboard viewer, users can mouse over the different view landmarks to reveal more information on the annotations.

The website features a Help section that includes interactive and instructive presentations. Each result section also provides a Help tab with specific explanations. All result pages feature an interactive Export menu for the download of selected raw data, as well as of the compiled archive with all data generated by the server. Additionally, we provide machine-readable output in XML and JSON. Output formatted for web presentations is available (HTML link at top right corner of main result page). The HTML view—most familiar to long-time users—aggregates results from most of the integrated methods in one page. This page also contains information that has not been integrated into the graphical view—yet—including results generated by some component methods and prediction confidence values. While we are working on the integration of all results into the graphical view, we highly encourage users to inspect this ‘raw’ HTML view. Finally, output is also available in text format (TEXT link, top right corner of results).

### PPcache: pre-calculated results versus interactive jobs

One of the most beneficial recent resources from PredictProtein is the PPcache—a database that currently holds pre-calculated results for 11.7 million unique proteins—including all proteins of model organisms. If pre-calculated results are available for a PredictProtein query in PPcache, these are immediately returned. For results older than three months, users are given the option to re-run the query, thereby updating the PPcache. If no result exists in the PPcache, the job is processed, and users are notified upon job completion. PPcache currently requires roughly 100TB of disk space. We plan to open this repository for public access through a specialized API.

### Downloadable software: packages and cloud-ready virtual machine

For full proteome analysis we make the full PredictProtein software suite available for download to be run either by installing the software packages on local machines or by deploying a virtual machine image in the cloud. Most methods from the PredictProtein pipeline are now available as open-source packages and are freely distributed through Debian (59) and Ubuntu. Following the Debian guidelines enforces best practices for software development and distribution and guarantees robustness, usability and maintainability of our software packages.

Users with access to cloud computing can download the PredictProtein Machine Image or PPMI (60), a disk image optimized for deployment in the cloud. The PPMI is bootable on server instances in cloud infrastructure services, or on locally installed virtualization software.

## USE CASE

We demonstrate the usability and properties of PredictProtein through a simple example, the human endoplasmatic reticulum (ER) membrane protein complex subunit 4 (EMC4, UniProt AC Q5J8M3; Figure 1B–E). EMC4 is a small alpha-helical transmembrane protein with 183 residues. It is relatively well annotated, localizes to the membrane of the ER and is implicated in apoptosis (61,62).

The dashboard view of PredictProtein reveals an N-terminal disordered region of ∼60 residues (Figure 1B) interrupted by a short beta-strand (residues 17–20). This mainly disordered region is followed by a region dominated by alpha-helices. In this region, two transmembrane helices are predicted. Note that mouse-over can reveal annotations. The lines below the predictions sketch proteins with similar sequence. EMC4 is highly conserved, and nearly identical proteins are found in several mammalian organisms. Interestingly, the heatmap of functional effects (SNAP2) shows that the beta-strand interrupting the N-terminal disordered region and the transmembrane helices are highly sensitive to point mutations (Figure 1C). LocTree3 and metastudent predictions, respectively, agree at high reliability with the experimental subcellular localization of EMC4 in the ER membrane and its function in apoptosis (61,62) (Figure 1D and E). Additionally, metastudent identifies ‘protein folding in endoplasmic reticulum’ as biological function (Figure 1D; directed graph of predicted GO terms in Supplementary Figure S1, Supporting Online Material). This has already been shown for the yeast EMC4 (63).

The EMC4 example shows how users could have suspected some of those findings that have been experimentally verified (transmembrane helices, apoptosis, ER localization). On the other hand, it also suggests additional insights that might trigger new experiments, e.g. the importance of the disordered N-terminus, and the importance of the beta-strand that breaks it. May be this will provide more detail on the suggested involvement in protein folding and in apoptosis (Figure 1D (62)).

## CONCLUSION

Over its 22 year existence, the PredictProtein server has substantially expanded. What started as a service to annotate some aspects of protein structure (secondary structure, solvent accessibility and transmembrane helices) has evolved into a comprehensive suite of methods important for the prediction of protein structural and functional features. It provides a single-point access to many original important results. Our focus on making reliable methods available and our technical focus on keeping our server useful to the community have sustained many challenges in an environment of low funding, growing use and increasing data deluge. Yet we continue finding ways to present our results efficiently and without overloading users from a wide variety of backgrounds and needs. The results pages aspire to give visually intuitive, unified presentations for most of the structural and functional annotations. The PredictProtein web server can help when little is known about the protein in question. For medium-to-high throughput analyses, users will find the publicly available, downloadable software packages and the PPMI a suitable option. For approximately every second query, our PPcache repository provides results immediately.

## SUPPLEMENTARY DATA

Supplementary Data are available at NAR Online.

Supplementary Data
