# Supplementary material for: PredictProtein—an open resource for online prediction of protein structural and functional features
Source: Nucleic Acids Res. 2014 May 5;42(Web Server issue):W337–43. doi: 10.1093/nar/gku366 (PMC4086098; doi:10.1093/nar/gku366)
Supplement: Supplementary Data [file supp_42_W1_W337__index.html]

Supplementary Data 

# PredictProtein—an open resource for online prediction of protein structural and functional features

## Supplementary Data

**Files in this Data Supplement:**

- SUPPLEMENTARY DATA
