# Supplementary material for: PredictProtein—an open resource for online prediction of protein structural and functional features
Source: Nucleic Acids Res. 2014 May 5;42(Web Server issue):W337–43. doi: 10.1093/nar/gku366 (PMC4086098; doi:10.1093/nar/gku366)
Supplement: Supplementary Data [file supp_gku366_nar-00497-web-b-2014-File003.pdf]

Supporting online material
for:
PredictProtein – open online prediction of protein structure and function

Guy Yachdav, Edda Kloppmann, Laszlo Kajan, Max Hecht, Tatyana Goldberg, Tobias Hamp, Andrea Schafferhans, Burkhard Rost et al

Table S1: Methods incorporated into PredictProtein

Category	Method/dB Name	Description	Command line tool		Output available in		
			Name	Version	Visual	HTML	TEXT
Database search	BLAST	Pairwise alignment	blastall	2.2.26	-	-	x
Database search	PSI-BLAST	Profile based alignment	blastpgp	2.2.27	x	x	x
Database search	HMMER	Hidden markov model search	hmm2pfam	2.3.2	-	-	x
Database search	ps_scan	PROSITE scanning program	ps_scan.pl	1.67	-	x	x
Database Search	PSSH	Mapping of sequence positions onto a structure	generate_pssh2	1.0.0	-	-	x
Database Search	Species Mapper	Maps organism code to kingdom	speciesmapper	1.0,1	x	-	-
Database Search	ID Mapper	maps ids across major databases	idmapper	1.0.3	x	-	-
Analysis method	SEG	Low complexity regions markup	ncbi-seg	0.0.20000620	-	x	x
Analysis method	NCOILS	Calculates the probability that the sequence will adopt a coiled-coil conformation	ncoils	2002	-	x	x
Analysis method	HSSP	Homology derived secondary Structure of proteins	hssp_filter	1	-	-	x
Prediction method	NORSp	NOn-Regular Secondary Structure	norsp	1.0.3	-	x	x
PHDhtm	PHDhtm	Prediction of membrane helices	phd.pl	1.0.40	x	x	x
Prediction method	TMSEG	Prediction of membrane helices	tmseg	1.0.0	x	x	x
Prediction method	PROFsec	Prediction of secondary structure state	prof	1.0.40	x	x	x
Prediction method	PROFacc	Prediction of solvent accessbility	prof	1.0.40	x	x	x
Prediction Method	Reprof	Improved prediction of secondary structure state	reprof	1.0.0	-	-	x
Prediction method	PROFtmb	Prediction of transmembrane beta-barrels	proftmb	1.1.12	x	x	x
Prediction method	DISULFIND	Prediction of disulfide bridges	disulfinder	1.2	x	x	x
Prediction method	PROFBval	Prediction of residue mobility	profbval	1.0.16	x	x	x

Category	Method/dB Name	Description	Command line tool		Output available in		
			Name	Version	Visu al	HTML	TEXT
Prediction method	NorsNet	Prediction protein disordered sites	norsnet	1.0.16	x	x	x
Prediction method	UCON	Contact based prediction of disordered sites	ucon	1.0.8	x	x	x
Prediction method	Meta-Disorder	Consensus based prediction of protein disorder	metadisorder	1.0.14	x	x	x
Prediction method	ISIS2	Prediction of protein-protein interaction sites	profisis2	1.0.0	x	x	x
Prediction method	SomeNA	Prediction of protein –DNA, -RNA binding sites	somena	1.0.0	X	-	-
Prediction method	LocTree3	Prediction of sub-cellular localization for all domains of life	loctree3	1.0.5	x	x	x
Prediction method	PredictNLS	Prediction of Nuclear Localization Signals (NLS)	predictnls	1.0.18	-	x	x
Prediction method	metastudent	Prediction of GO terms for Molecular Function and Biological Process	metastudent	1.0.9	x	-	X
Prediction method	SNAP2	Prediction of functional changes due to single nucleotide polymorphism	snap2	1.0.10	x	-	x
Prediction method	ConSurf	Identification of functional sites	consurf	1.0.0	x	-	x
Database	UniRef	Clustered set of sequences	N/A	Updates monthly	x	x	X
Database	BIG	non-redundant combination of Swiss-Prot, TrEMBL, PDB	N/A	Updates monthly	x	x	x
Database	PDB	Repository of protein structures	N/A	Updates monthly	x	x	x
Database	Pfam-A	Protein families	N/A	Updates quarterly	x	x	x
Database	PROSITE	Database of biologically significant sites, patterns and profiles	N/A	Updates quarterly	x	x	X

Table S2: List of contributors

This table lists all non-coauthors contributors. All contributors are acknowledged at http://ppopen.rostlab.org/credits

Name	Contribution	Affiliation
Jinfeng Liu	Contributed code for the PredictProtein pipeline<br>Contributed the NORS, CHOP & CHOPnet (discontinued) methods	Alumnus
Yanay Ofran	Contributed the ISIS and DISIS methods (discontinued)	Alumnus
Rajesh Nair	Contributed the LocTree method (discontinued)	Alumnus
Henry Bigelow	Contributed the PROFtmb method	Alumnus
Sven Mika	Provided the UniqueProt method	Alumnus
Dariusz Przybylski	Contributed the AGAPE method (discontinued)	Alumnus
Kazimierz Wrzeszczynski	Contributed code and ideas	Alumnus
Paolo Frasconi	Contributed the DISULFIND method	External contributor
Antoine de Daruvar	Helped getting the first PredictProtein server online	Original contributor
Roy Omond	Helped in the communication between VMS and Unix systems for the first server	Original contributor
Jonas Reeb	Member of the Scientific Editorial Board<br>Responsible for transmembrane annotations	Scientific board
Juan Miguel Cejuela	Contributed the literature search component	Scientific board
Rachel First	Designed the artwork for the localization prediction<br>Designed the site tutorial	Graphics design
Thomas Splettstoesser	Designed the PredictProtein logo	Graphics design

Figure S1

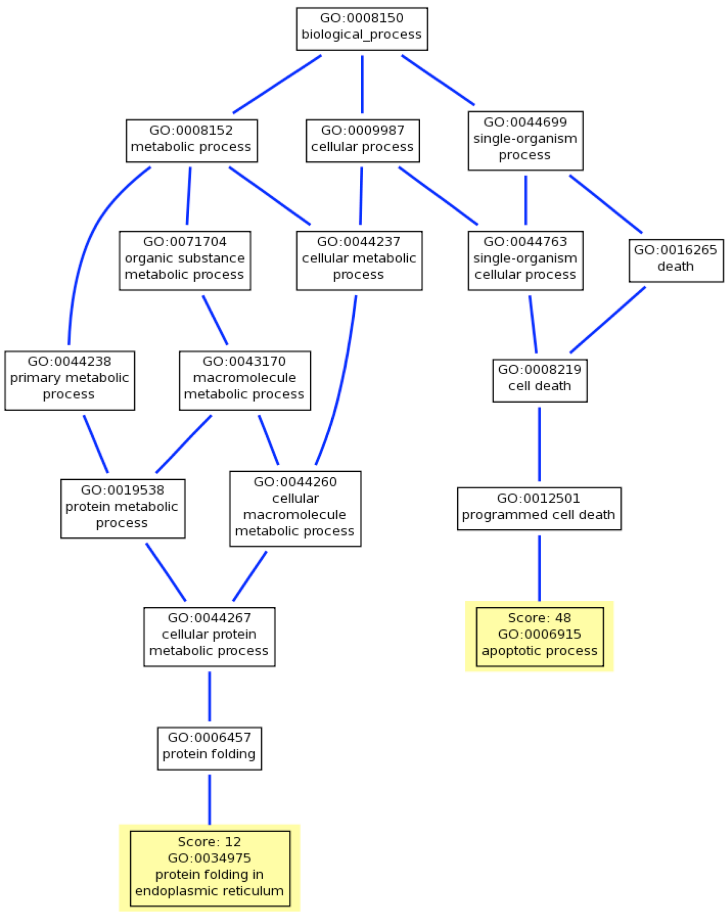

Figure S1: GO term predictions. GO term predictions from metastudent are presented in PredictProtein in tabular form (Fig. 1D) and as directed graph as shown here through the example of human EMC4 (UniProt AC Q5J8M3).
